# Associations between renal function, hippocampal volume, and cognitive impairment in 544 outpatients

**DOI:** 10.3389/fneur.2024.1347682

**Published:** 2024-06-04

**Authors:** Lei-Yun Wu, Yuan-Yuan Lu, Shuang-Shuang Zheng, Ya-Dong Cui, Jie Lu, Ai-Hua Zhang

**Affiliations:** ^1^Department of Nephrology, Xuanwu Hospital, Capital Medical University, Beijing, China; ^2^Department of Neurology and Innovation Center for Neurological Disorders, Xuanwu Hospital, Capital Medical University, National Center for Neurological Disorders, Beijing, China; ^3^Department of Radiology and Nuclear Medicine, Xuanwu Hospital, Capital Medical University, Beijing, China; ^4^Department of Radiology, Fuxing Hospital, Capital Medical University, Beijing, China; ^5^Beijing Key Laboratory of Magnetic Resonance Imaging and Brain Informatics, Beijing, China; ^6^National Clinical Research Center for Geriatric Disorders, Xuanwu Hospital, Capital Medical University, Beijing, China

**Keywords:** renal function, hippocampus volume, estimated glomerular filtration, cognitive impairment, magnetic resonance imaging

## Abstract

**Background:**

Cognitive impairment and brain atrophy are common in chronic kidney disease patients. It remains unclear whether differences in renal function, even within normal levels, influence hippocampal volume (HCV) and cognition. We aimed to investigate the association between estimated glomerular filtration rate (eGFR), HCV and cognition in outpatients.

**Methods:**

This single-center retrospective study enrolled 544 nonrenal outpatients from our hospital. All participants underwent renal function assessment and 3.0 T magnetic resonance imaging (MRI) in the same year. HCV was also measured, and cognitive assessments were obtained. The correlations between eGFR, HCV, and cognitive function were analyzed. Logistic regression analysis was performed to identify the risk factors for hippocampal atrophy and cognitive impairment. Receiver-operator curves (ROCs) were performed to find the cut-off value of HCV that predicts cognitive impairment.

**Results:**

The mean age of all participants was 66.5 ± 10.9 years. The mean eGFR of all participants was 88.5 ± 15.1 mL/min/1.73 m^2^. eGFR was positively correlated with HCV and with Mini-Mental State Examination (MMSE) and Montreal Cognitive Assessment (MoCA) scores. Univariate and multivariate logistic regression analysis showed Age ≥ 65 years, eGFR < 75 mL/min/1.73 m^2^, Glucose ≥6.1 mmol/L and combined cerebral microvascular diseases were independent risk factors for hippocampal atrophy and Age ≥ 65 years, left hippocampal volume (LHCV) <2,654 mm^3^ were independent risk factors for cognitive impairment in outpatients. Although initial unadjusted logistic regression analysis indicated that a lower eGFR (eGFR < 75 mL/min/1.73 m^2^) was associated with poorer cognitive function, this association was lost after adjusting for confounding variables. ROC curve analysis demonstrated that LHCV <2,654 mm^3^ had the highest AUROC [(0.842, 95% CI: 0.808–0.871)], indicating that LHCV had a credible prognostic value with a high sensitivity and specificity for predicting cognitive impairment compared with age in outpatients.

**Conclusion:**

Higher eGFR was associated with higher HCV and better cognitive function. eGFR < 75 mL/min/1.73 m^2^ was an independent risk factor for hippocampal atrophy after adjusting for age. It is suggested that even eGFR < 75 mL/min/1.73 m^2^, lower eGFR may still be associated with hippocampal atrophy, which is further associated with cognitive impairment. LHCV was a favorable prognostic marker for predicting cognitive impairment rather than age.

## Introduction

Cognitive impairment is common in chronic kidney disease (CKD) (1, 2), and poor renal function has been associated with cognitive impairment in CKD patients (3). Patients with CKD are significantly more likely to have disproportionate levels of cerebrovascular disease, particularly small-vessel cerebrovascular disease, suggesting that these may be important factors in the development of CKD-related cognitive impairment (4). A study of 1,527 community-based participants showed that kidney function measures are significantly associated with markers of neurodegeneration and small vessel disease visible on brain MRI (5). Current studies believe that CKD-related microvascular injury may be the main factor leading to cognitive impairment (4). There are multifaceted evidence supporting this hypothesis. First, individuals with CKD have a high prevalence of cardiovascular disease (CVD) and CVD risk factors, including diabetes, hypertension, and dyslipidemia, frequently severe enough to lead to renal failure. Second, those with CKD are more susceptible to clinical cerebrovascular disease, including stroke and transient ischemic attack, as well as have subclinical cerebrovascular disease on imaging such as small-vessel infarcts, lacunes, and white matter disease. Third, the cognitive impairment associated with cerebrovascular disease primarily affect processing and executive function, cognitive domains that affect planning and carrying out tasks, and most studies found that processing speed and executive function are the domains most affected in individuals with CKD. Fourth, CVD and its risk factors are associated with worse executive function. Moreover, in earlier stages of CKD, the presence of albuminuria, possible representative of systemic vascular injury, is associated with worse executive function and incident dementia (4). Cognitive impairment in CKD is influenced by multifactor and is accompanied by brain atrophy, including hippocampal atrophy (6, 7). Reduced hippocampal volume (HCV) is associated with late-life cognitive decline (8). HCV is usually defined as an imaging marker of cognitive impairment in general population (8). Most previous studies on the relationship between eGFR and HCV paid attention to patients with clinically significant renal injury (e.g., eGFR < 60 mL/min/1.73 m^2^) (9, 10). A study found that eGFR < 60 mL/min/1.73 m^2^ was significantly associated with cortical atrophy in nondisabled adults (11). A three-year observation in elderly diabetic patients found that changes in eGFR during follow-up were independent risk factors for hippocampal atrophy (mean eGFR decreased from 65 mL/min/1.73 m^2^ to 59 mL/min/1.73 m^2^) (12). However, is there a relationship between renal function and HCV in nonrenal disease population? Reports about this topic are scarce, and the results are controversial (13, 14). Two studies from the ADNI database came to different conclusions: a cohort study composed of all participants with eGFR > 60 mL/min/1.73 m^2^ found that higher renal function was associated with slower hippocampal atrophy and cognitive decline even within the normal range of renal function (13). Another cross-sectional study found that a mildly to moderately reduced eGFR (eGFR > 45 mL/min/1.73 m^2^) was not associated with brain atrophy in ADNI participants (14). Dementia and mild cognitive impairment (MCI) are highly prevalent in China (15). There are fewer studies on the relationship between GFR, HCV, and cognitive function in the real world. In this study, we will explore the association between eGFR, HCV, and cognitive function in outpatients who are not from a renal clinic.

## Materials and methods

### Study population

**Figure 1:**
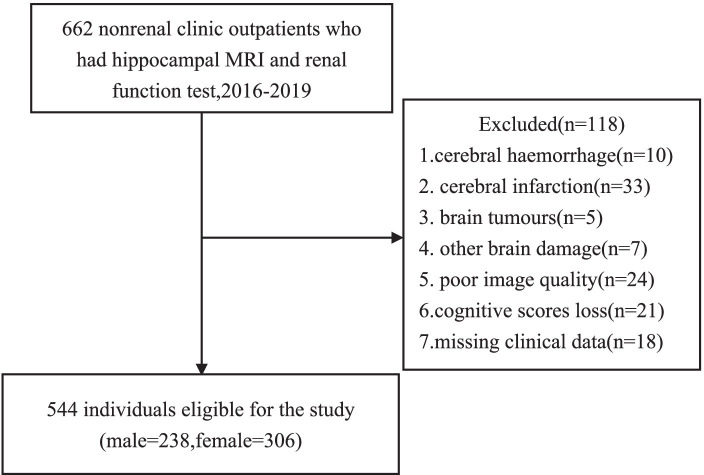
Flow chart on selection of participants.

This was a cross-sectional study of the majority of outpatients with normal renal function recruited from the nonrenal clinic of Xuanwu Hospital, Capital Medical University. Participants who were undergoing 3.0 T MRI with oblique coronal thin sections oriented perpendicular to the hippocampal long axis were consecutively recruited from Jan 2016 to Dec 2019. The inclusion criteria were as follows: (1) patients with renal function results in the same year (5). The exclusion criteria were as follows: (1) patients with apparent brain lesions, such as cerebral hemorrhage, cerebral infarction, brain tumor, and other brain damage; (2) patients with poor image quality; and (3) patients without MMSE and MoCA scores. A total of 662 outpatients met the inclusion criteria, among whom 118 were excluded from this estimate, including 10 with cerebral hemorrhage, 33 subjects with cerebral infarction, 5 with brain tumors, 7 with other brain damage, 24 with poor image quality, 21 without MMSE and MoCA scores and 18 with missing clinical data. Thus, a total of 544 subjects were recruited (Figure 1). Among them, 325 cases were diagnosed with cognitive impairment, including 8 cases were diagnosed with AD, 31 cases were diagnosed with dementia, 17 cases were diagnosed with vascular dementia. 219 cases were cognitively normal.

### Clinical characteristics and laboratory data

Patient characteristics included age, sex, clinical diagnosis, MRI diagnosis, and laboratory data including serum urea, serum creatinine, total protein, albumin, and serum uric acid (SUA), glucose, total cholesterol, low density lipoprotein, triglyceride, homocysteine, potassium, phosphate, alanine aminotransferase (ALT), leukocytes, hemoglobin, platelets. Serum creatinine concentrations were measured by an automatic biochemical analyzer (BioTek Instrument, Inc., Beijing, China) and used to compute eGFR based on the CKD-Epidemiology Collaboration (CKD-EPI) equation (16). We chose the CKD-EPI equation as it is more accurate in the elderly and at higher levels of kidney function (eGFR > 60 mL/min/1.73 m^2^) and is a better predictor of mortality when compared to the Modification of Diet in Renal Disease Study equation (17–19). The CKD-EPI equation (16) was calculated as recommended: For women with a plasma creatinine ≤0.7 mg/dL,(plasma creatinine/0.7)^−0.329^ × (0.993)^age^ (×166 if black; ×144 if white or other); for women with a plasma creatinine >0.7 mg/dL, (plasma creatinine/0.7)^−1.209^ × (0.993)^age^ (×166 if black; ×144 if white or other); for men with a plasma creatinine ≤0.9 mg/dL, (plasma creatinine/0.9)^−0.411^ × (0.993)^age^ (×163 if black; ×141 if white or other),for men with a plasma creatinine >0.9 mg/dL, (plasma creatinine/0.9)^−1.209^ (×0.993)^age^ (×163 if black; ×141 if white or other).

### MRI acquisition and analysis

All MR images were obtained on a 3.0 T MR scanner (GE DISCOVERY 750, America) with a 20-channel coil. All participants underwent MRI with oblique coronal thin sections oriented perpendicular to the hippocampal long axis. The scanning parameters were as follows: For Cor T1 FSPGR scanning, we used Fast Spoiled Gradient Recalled sequence, repetition time (TR) = 115 ms, echo time (TE): 2.1 ms, Flip Angle:70°, layer thickness = 3 mm, matrix size = 2,256 × 192, and layer number = 23.

All MRI images were evaluated by two experienced radiologists to exclude patients with obvious brain lesions and poor image quality.

### Hippocampal volume calculation

**Figure 2:**
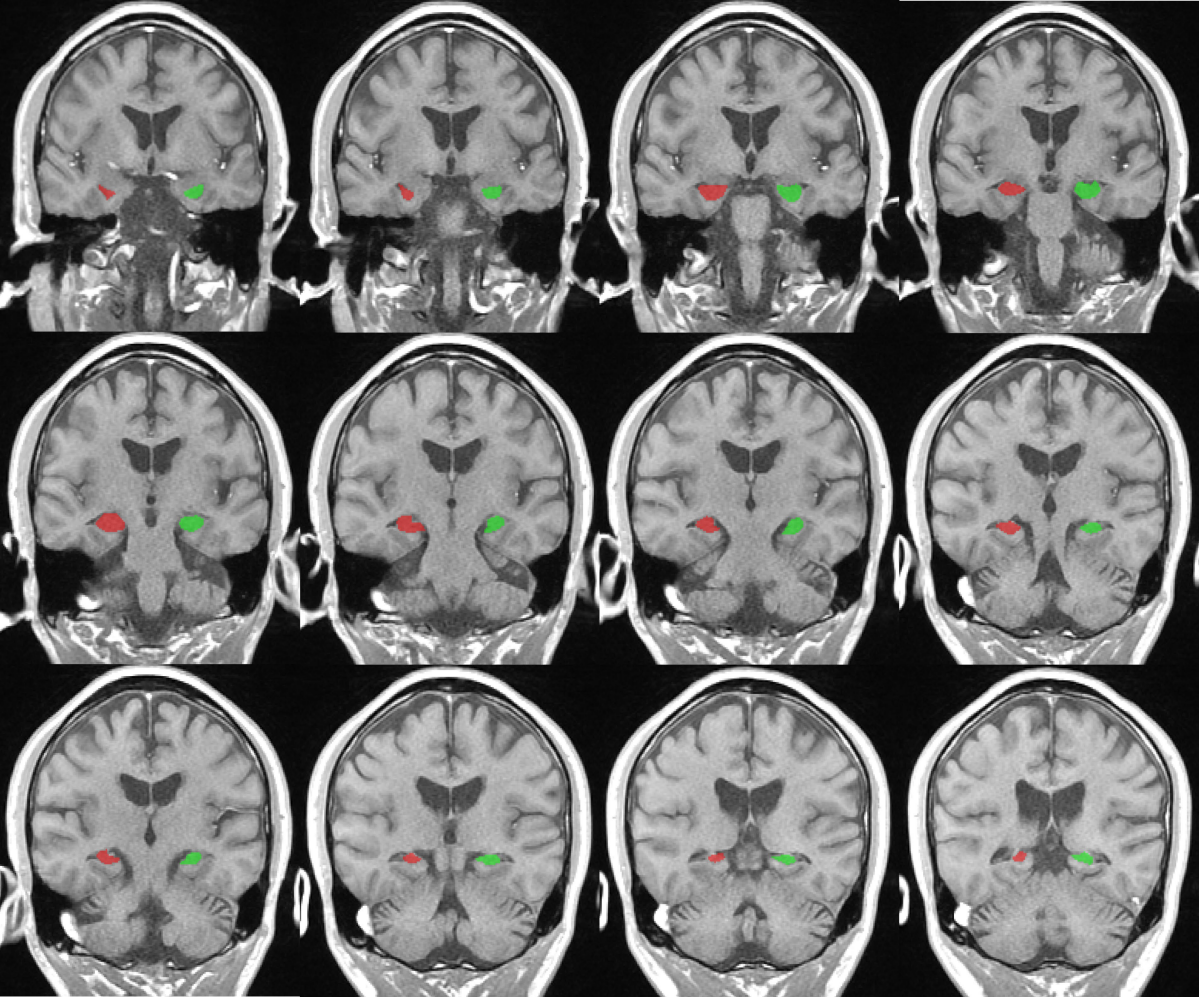
Representative example of right (red) and left (green) hippocampus segmentation and volume calculation on T1-weighted images in ITK-snap software.

T1-weighted structural images were analyzed for the measurement of hippocampal volume. First, we manually segmented the hippocampus and identified the boundaries of the hippocampus through the sagittal and coronal plane and drew an outline of the structure of the hippocampus (20, 21). The critical point of hippocampal volume measurement is to accurately calibrate the oblique coronal section perpendicular to the long axis of the hippocampus and the oblique coronal section from the head to the tail of the hippocampus as the main measurement section (20, 21). Then, the ITK-SNAP software was used to delineate the boundary of the hippocampal structure, with oblique coronal images serving as the reference level and horizontal and sagittal images aiding in boundary identification (Figure 2). Subsequently, the software automatically computed the volume of the hippocampus (22). Finally, we computed the intracranial volume (ICV) and obtained standardization via dividing hippocampal volume by total brain volume (23). Two proficient investigators independently measured bilateral hippocampal volumes and cross-validated their findings.

### Cognitive function

All patients had a medical history in the outpatient department of neurology, and MMSE and MoCA scores were recorded. MoCA score < 26 was defined as cognitive impairment (24).

### Cerebral microvascular diseases

The presence of cerebral microvascular diseases was defined as microbleeds, microinfarcts, vascular spaces, and increased white matter lesions in MRI reports.

### Statistical analysis

Statistical analysis was done by using SPSS version 23.0 (IBM) and Med Calc version 20.116. The Kolmogorov–Smirnov test was used to evaluate the distribution of the variables. For continuous variables, the data was expressed as mean ± SD or median (interquartile range). Categorical variables were displayed as percentages or ratios. The Student t-test, Mann–Whitney U test or Chi-square test was performed for comparisons between variables at the right time. Spearman correlation analysis was performed between eGFR and HCV as well as eGFR and cognitive function. ROCs were utilized to evaluate the predictive sensitivity and specificity of different markers for cognitive impairment, and the areas under the curves (AUCs) were computed. All analyses were two-tailed, and statistical significance was accepted at *p* < 0.05.

## Results

### Comparison of clinical characteristics and HCV between hippocampal atrophy patients and nonhippocampal atrophy patients

MTA visual scale was used to make the imaging diagnosis of hippocampal atrophy in the Radiology department of our hospital. A total of 544 participants were divided into two groups: 226 in the hippocampal atrophy group and 318 in the nonhippocampal atrophy group. The baseline demographic and clinical characteristics are shown in Table 1. There were no significant differences in sex, combined hypertension, combined diabetes, total protein, SUA, total cholsterol, low density lipoprotein, triglycerides, potassium, phosphate, leukocytes, platelet counts between the two groups. Compared with patients in the nonhippocampal atrophy group, patients in the hippocampal atrophy group were older [70.8 ± 9.6 years vs. 62.4 ± 10.8 years, (*p* < 0.001)], and were vulnerable to cerebral microvascular diseases [67.3% vs. 33.6%, (*p* < 0.001)]. In terms of laboratory findings, patients in the hippocampal atrophy group had higher levels of serum urea, serum creatinine, glucose, and homocysteine, but lower levels of eGFR, albumin, ALT and hemoglobin. Notably, mean eGFR of both groups were within the normal levels [84.7 ± 15.3 mL/min/1.73 m^2^ vs. 91.2 ± 14.4 mL/min/1.73 m^2^, (*p* < 0.001)].

**Table 1:** Comparison of baseline demographic, clinical characteristics, and combined cerebral microvascular diseases between hippocampal atrophy patients and nonhippocampal atrophy patients.

	Hippocampal atrophy (*n* = 226)	Nonhippocampal atrophy (*n* = 318)	*p*-value
Age (years)	70.8 ± 9.6	62.4 ± 10.8	<0.001
Male (%)	106 (46.9%)	132 (41.5%)	0.211
Hypertension (%)	112 (49.6%)	156 (49.1%)	0.908
Diabetes (%)	121 (53.5%)	167 (52.5%)	0.814
Serum urea (mmol/L)	5.9 ± 1.8	5.6 ± 1.4	0.009
Serum creatinine (μmol/L)	65.8 ± 17.5	60.4 ± 12.6	<0.001
eGFR (mL/min/1.73 m^2^)	84.7 ± 15.3	91.2 ± 14.4	<0.001
Total protein (g/L)	69.9 ± 4.5	70.4 ± 4.3	0.244
Albumin (g/L)	42.4 ± 3.0	43.5 ± 2.8	<0.001
SUA (mmol/L)	318.4 ± 90.6	315.7 ± 79.5	0.781
Glucose (mmol/L)	5.5 (5.2, 6.3)	5.4 (4.9, 5.8)	<0.001
Total cholesterol (mg/dL)	4.8 ± 1.1	4.7 ± 0.9	0.362
Low density lipoprotein (mmol/L)	2.9 ± 0.9	2.9 ± 0.8	0.698
Triglyceride (mmol/L)	1.2 (0.9, 1.6)	1.3 (0.9, 1.8)	0.237
Homocysteine (μmol/L)	13.9 (11.4, 18.1)	12.5 (10.6, 15.3)	<0.001
Potassium (mmol/L)	4.4 ± 0.4	4.4 ± 0.3	0.563
Phosphate (mmol/L)	1.1 ± 0.1	1.1 ± 0.2	0.491
ALT (IU/L)	16 (12, 22)	19 (15, 29)	0.005
Leukocytes (×10^9^/L)	6.0 ± 1.6	5.8 ± 1.5	0.213
Hemoglobin (g/L)	138.2 ± 15.4	143.1 ± 14.9	0.004
Platelets (×10^9^/L)	206.4 ± 52.7	211.7 ± 52.3	0.353
Cerebral microvascular diseases (%)	152 (67.3%)	107 (33.6%)	<0.001
LHCV (mm^3^)	1797 (1,125, 1,679)	3,097 (2,756, 3,361)	<0.001
RHCV (mm^3^)	1,627 (1,311, 1,968)	3,009 (2,714, 3,278)	<0.001
Left hippocampal height (mm)	6.6 (5.7, 7.5)	8.1 (7.4, 8.5)	<0.001
Right hippocampal height (mm)	6.4 (5.6, 7.3)	7.9 (7.3, 8.4)	<0.001
Left width of choroid fissure (mm)	4.1 (2.8, 5.0)	1.7 (1.6, 1.9)	<0.001
Right width of choroid fissure (mm)	4.2 (2.8, 5.2)	1.8 (1.6, 2.0)	<0.001
Left width of temporal horn (mm)	5.0 (3.6, 6.7)	1.8 (1.6, 2.1)	<0.001
Right width of temporal horn (mm)	4.2 (2.7, 5.6)	1.9 (1.7, 2.2)	<0.001
MMSE (score)	24 (16, 27)	28 (26, 30)	<0.001
MoCA (score)	18 (10, 23)	26 (22, 30)	<0.001

eGFR, estimated glomerular filtration rate; SUA, serum uric acid; ALT, alanine aminotransferase; LHCV, left hippocampal volume; RHCV, right hippocampal volume; MMSE, Mini-Mental State Examination; MoCA, Montreal Cognitive Assessment.

### Risk factors for hippocampal atrophy

Univariate logistic regression demonstrated that age ≥ 65 years, eGFR < 75 mL/min/1.73 m^2^, Glucose≥6.1 mmol/L, homocysteine≥15 μmol/L, combined cerebral microvascular diseases were the risk factors for hippocampal atrophy; whereas multivariate logistic regression analysis showed that age ≥ 65 years, eGFR < 75 mL/min/1.73 m^2^, Glucose≥6.1 mmol/L and combined cerebral microvascular diseases were independent risk factors for hippocampal atrophy (Table 2).

**Table 2:** Univariate analysis and multivariate analysis of prognostic factors for hippocampal atrophy.

Characteristic	Univariate analysis			Multivariate analysis		
	HR	95% CI	*p*-value	HR	95% CI	*p*-value
Age ≥ 65 years	3.268	2.263 ~ 4.720	<0.001	3.194	1.883 ~ 5.416	<0.001
eGFR<75 mL/min/1.73 m^2^	2.759	1.680 ~ 4.530	<0.001	2.474	1.619 ~ 3.780	<0.001
Albumin < 35 g/L	0.895	0.556 ~ 1.440	0.648	-	-	-
Glucose ≥ 6.1 mmol/L	1.738	1.165 ~ 2.593	0.007	1.757	1.067 ~ 2.892	0.027
Homocysteine ≥ 15 μmol/L	1.704	1.167 ~ 2.488	0.006	1.474	0.955 ~ 2.275	0.080
ALT<40 IU/L	1.596	0.765 ~ 3.327	0.213			
Hemoglobin<120 g/L	1.009	0.714 ~ 1.425	0.961	-	-	-
Cerebral microvascular diseases	5.097	3.540 ~ 7.340	<0.001	4.835	3.172 ~ 7.368	<0.001

eGFR, estimated glomerular filtration rate; ALT, alanine aminotransferase.

### Comparison of clinical characteristics and HCV between cognitive impairment patients and cognitively normal patients

MoCA score < 26 was defined as the threshold of cognitive impairment and 544 outpatients were divided into two groups: the cognitive impairment group and cognitively normal group. As shown in Table 3, patients with cognitive impairment had lower eGFR than patients without cognitive impairment[87.3 ± 12.7 mL/min/1.73 m^2^ vs. 92.2 ± 15.3 mL/min/1.73 m^2^, (*p* < 0.001)]. Patients with cognitive impairment had significantly smaller HCV, lower hippocampal height, wider choroid fissure and temporal horn than patients without cognitive impairment (*p* < 0.001); they showed older age and lower albumin level than those without cognitive impairment (*p* < 0.001). There were significant differences in SUA, glucose, triglyceride, homocysteine, ALT and hemoglobin between the two groups (*p* < 0.05). There were more patients with cerebral microvascular diseases in the cognitive impairment group than in the cognitively normal group (*p* < 0.05). There were no significant differences in sex, combined hypertension, combined diabetes, serum urea, serum creatinine, total protein, total cholesterol, low density lipoprotein, potassium, phosphate, leukocytes and platelets between the two groups (Table 3).

**Table 3:** Comparison of baseline demographic, clinical characteristics, combined cerebral microvascular diseases, and hippocampal measurements between cognitive impairment patients and cognitively normal patients.

	Cognitive impairment group (*n* = 325)	Cognitively normal group (*n* = 219)	*p*-value
Male (%)	132 (40.6%)	106 (48.4%)	0.073
Age (years)	70.2 ± 9.4	60.8 ± 10.5	<0.001
Hypertension (%)	167 (53.0%)	110 (50.5%)	0.561
Diabetes (%)	173 (54.9%)	106 (48.6%)	0.152
Serum urea (mmol/L)	5.8 ± 1.7	5.6 ± 1.3	0.197
Serum creatinine (μmol/L)	61.2 ± 13.0	62.1 ± 13.0	0.453
eGFR (mL/min/1.73 m^2^)	87.3 ± 12.7	92.2 ± 15.3	<0.001
Total protein (g/L)	69.9 ± 4.3	70.5 ± 4.3	0.148
Albumin (g/L)	42.6 ± 3.0	43.9 ± 2.7	<0.001
SUA (mmol/L)	305.8 ± 77.6	326.8 ± 85.6	0.027
Glucose (mmol/L)	5.5 (5.1, 6.2)	5.3 (4.9, 5.7)	0.001
Total cholesterol (mg/dL)	4.8 ± 1.0	4.7 ± 0.9	0.520
Low density lipoprotein (mmol/L)	2.9 ± 0.9	2.8 ± 0.8	0.210
Triglyceride (mmol/L)	1.2 (0.8, 1.6)	1.3 (0.9, 1.8)	0.016
Homocysteine (μmol/L)	13.5 (11.1, 16.0)	12.5 (10.5, 15.5)	0.040
Potassium (mmol/L)	4.4 ± 0.4	4.4 ± 0.3	0.980
Phosphate (mmol/L)	1.1 ± 0.1	1.1 ± 0.2	0.849
ALT (IU/L)	16 (12, 22)	19 (15, 29)	<0.001
Leukocytes (×10^9^/L)	5.9 ± 1.5	5.9 ± 1.5	0.646
Hemoglobin (g/L)	138.8 ± 15.1	145.3 ± 14.6	<0.001
Platelets (×10^9^/L)	209.5 ± 53.6	210.1 ± 51.4	0.927
Cerebral microvascular diseases (%)	145 (46.0%)	43 (19.7%)	<0.001
LHCV (mm^3^)	1738 (1,242, 2,756)	3,143 (2,761, 3,426)	<0.001
RHCV (mm^3^)	1985 (1,451, 2,739)	3,022 (2,663, 3,298)	<0.001
Left hippocampal height (mm)	7.1 (6.0, 7.8)	8.2 (7.6, 8.6)	<0.001
Right hippocampal height (mm)	7.0 (5.9, 7.8)	8.1 (7.5, 8.5)	<0.001
Left width of choroid fissure (mm)	2.7 (1.9, 4.5)	1.7 (1.6, 1.9)	<0.001
Right width of choroid fissure (mm)	2.9 (1.9, 4.6)	1.8 (1.6, 2.0)	<0.001
Left width of temporal horn (mm)	3.5 (2.1, 5.7)	1.8 (1.6, 2.1)	<0.001
Right width of temporal horn (mm)	2.8 (2.0, 4.9)	1.9 (1.7, 2.2)	<0.001
MMSE (score)	24 (17, 26)	30 (28, 30)	<0.001
MoCA (score)	18 (11, 22)	28 (26, 30)	<0.001

eGFR, estimated glomerular filtration rate; SUA, serum uric acid; ALT, alanine aminotransferase; LHCV, left hippocampal volume; RHCV, right hippocampal volume; MMSE, Mini-Mental State Examination; MoCA, Montreal Cognitive Assessment.

### Association between eGFR and HCV with cognitive function

**Figure 3:**
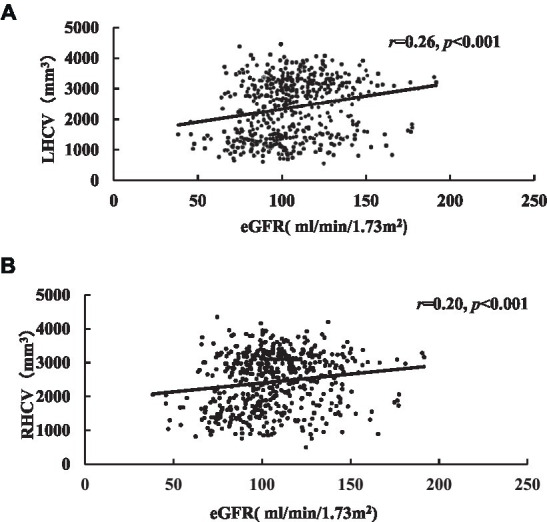
Correlation analysis of eGFR related to LHCV **(A)** and RHCV **(B)**. eGFR, estimated glomerular filtration rate; LHCV, left hippocampal volume; RHCV, right hippocampal volume.

HCV were measured and cognitive assessment were obtained in 544 patients. The medians of LHCV and right hippocampal volume (RHCV) were 2,587 mm^3^ and 2,600 mm^3^, respectively. eGFR was positively correlated with HCV and cognitive function (Table 4; Figure 3).

**Table 4:** Spearman correlation analysis of eGFR with hippocampal measurements and cognitive function.

Variable	eGFR (mL/min/1.73 m^2^)	
	Correlation Coefficient	*p*-value
LHCV (mm^3^)	0.244	<0.001
RHCV (mm^3^)	0.164	<0.001
Left hippocampal height (mm)	0.125	0.003
Right hippocampal height (mm)	0.122	0.004
Left width of choroid fissure (mm)	−0.228	<0.001
Right width of choroid fissure (mm)	−0.220	<0.001
Left width of temporal horn (mm)	−0.221	<0.001
Right width of temporal horn (mm)	−0.190	<0.001
MMSE (score)	0.143	0.001
MoCA (score)	0.148	0.001

eGFR, estimated glomerular filtration rate; LHCV, left hippocampal volume; RHCV, right hippocampal volume; MMSE, Mini-Mental State Examination; MoCA, Montreal Cognitive Assessment.

### Risk factors for cognitive impairment

Although univariate logistic regression suggested that eGFR < 75 mL/min/1.73 m^2^, glucose ≥6.1 mmol/L, combined cerebral microvascular diseases, LHCV < 2,654 mm^3^ and RHCV < 2,225 mm^3^ were risk factors for cognitive impairment, but multivariate logistic regression analysis showed that age ≥ 65 years, LHCV < 2,654 mm^3^ were independent risk factors for cognitive impairment (Table 5).

**Table 5:** Univariate analysis and multivariate analysis of prognostic factors for cognitive impairment patients.

Characteristic	Univariate analysis			Multivariate analysis		
	HR	95% CI	*P* value	HR	95% CI	*p*-value
Age ≥ 65 years	3.896	2.717 ~ 5.586	<0.001	2.119	1.342 ~ 3.346	0.001
eGFR<75 mL/min/1.73 m^2^	2.034	1.202 ~ 3.442	0.008	0.884	0.458 ~ 1.707	0.713
Albumin<35 g/L	1.292	0.799 ~ 2.088	0.296	-	-	-
SUA < 420 mmol/L	1.502	0.727 ~ 3.105	0.272	-	-	-
Glucose ≥ 6.1 mmol/L	2.178	1.415 ~ 3.352	<0.001	1.629	0.963 ~ 2.755	0.069
Triglyceride<0.45 mmol/L	1.040	0.390 ~ 2.774	0.938	-	-	-
Homocysteine ≥ 15 μmol/L	1.334	0.907 ~ 1.962	0.143	-	-	-
ALT<40 IU/L	1.691	0.850 ~ 3.364	0.135	-	-	-
Hemoglobin<120 g/L	2.421	0.779 ~ 7.525	0.126	-	-	-
Cerebral microvascular diseases	3.473	2.425 ~ 4.973	<0.001	1.474	0.929 ~ 2.339	0.099
LHCV<2,654 mm^3^	13.143	8.645 ~ 19.981	<0.001	6.345	3.197 ~ 12.592	<0.001
RHCV<2,225 mm^3^	10.681	6.994 ~ 16.311	<0.001	1.861	0.915 ~ 3.788	0.087

eGFR, estimated glomerular filtration rate; SUA, serum uric acid; ALT, alanine aminotransferase; LHCV, left hippocampal volume; RHCV, right hippocampal volume.

### Predictive accuracy of different markers for cognitive impairment in outpatients

**Figure 4:**
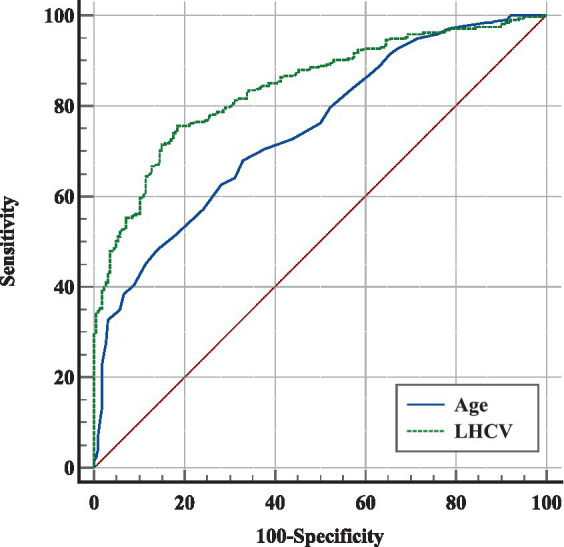
ROC analysis of different markers in predicting cognitive impairment in outpatients. The ROCs of different markers showed that LHCV had the largest AUC and a credible prognostic value with a high sensitivity and specificity for predicting cognitive impairment in outpatients. LHCV, left hippocampal volume.

In Figure 4, ROCs were obtained from the risk factors that were statistically significant in our previous analysis. LHCV showed the largest AUC of 0.842 and demonstrated more significant diagnostic value than age (*p* < 0.001). The optimal LHCV threshold was 2,654 mm^3^ for predicting cognitive impairment in outpatients.

## Discussion

In contrast to other studies, our study was a retrospective cross-sectional study of the relationship between renal function, HCV, and cognitive function in outpatients who were not from the nephrology department. We found that eGFR was positively correlated with HCV and cognitive function. The results are consistent with other studies (9, 25). We found that eGFR <75 mL/min/1.73 m^2^ was an independent risk factor for hippocampal atrophy. Although univariate logistic regression suggested that eGFR <75 mL/min/1.73 m^2^ was a risk factor for cognitive impairment, the link disappeared after multivariate analysis. Our study found that a smaller LHCV was an independent risk factor for cognitive impairment. LHCV <2,654 mm^3^ can predict cognitive impairment in outpatients (Figure 4).

HCV is a recognized imaging marker of cognitive dysfunction (8). The faster volume loss of the hippocampus suggested a higher risk of disease progression in mild cognitive impairment patients (26). Compared with the normal population, patients with CKD frequently had hippocampal atrophy (9), The possible cause is the damage of hippocampal cells caused by uraemic toxin (27). But, most previous studies concerned about participants with eGFR <60 mL/min/1.73 m^2^. However, in the majority participants with normal renal function, there are few studies on the relationship between renal function, HCV and cognitive function (12–14).

We found that eGFR was positively correlated with HCV and cognitive function, which was consistent with most studies (9, 28). We found age ≥ 65 years was an independent risk factor for cognitive impairment and hippocampal atrophy. The strong association between age and brain volume as well as cognitive function is not unexpected (29). We found hyperglycemia was an independent risk factor for hippocampal atrophy, which was similar to other studies (30, 31). Additionally, we also found combined cerebral microvascular diseases was another independent risk factor for hippocampal atrophy (32).

In our current study, we found that eGFR <75 mL/min/1.73 m^2^ was an independent risk factor for hippocampal atrophy although it is within defined normal renal function after adjusting for age. Studies on the relationship between eGFR and HCV in people within normal renal function are few and results are controversial (13, 14). For example, two studies from the same database (ADNI) at different times produced two different conclusions: An H, et al. reported that a longitudinal study including 1,269 patients with eGFR >60 mL/min/1.73 m^2^ found that higher renal function was associated with slower cognitive decline and hippocampal atrophy even within the range of normal renal function (13). However, another cross-sectional study including 1,596 patients (73% of patients with eGFR >60 mL/min/1.73 m^2^) found that a mild to moderately reduced eGFR (eGFR >45 mL/min/1.73 m^2^) was not associated with hippocampal atrophy (14). However, even in the study (14) also found that eGFR >90 mL/min 1.73 m^2^ was associated with larger brain volumes including HCV. A decline in eGFR accelerated age-related changes in brain volume (33). Only 2.0% of the patients in our study with GFR < 60 mL/min/1.73 m^2^, and the basic composition was similar to An. H’s study (13).

Although the mechanisms of the pathological association between kidney function and hippocampal atrophy remain unclear, several hypotheses might explain this correlation. First, a path analysis model suggested that vascular load play an important role in hippocampal atrophy even within normal ranges of renal function (13). Additionally, vascular burden induced amyloid deposition, which also linked with hippocampal atrophy (13). Most participants in our study had hypertension, diabetes or both diseases, which could generate vascular burden (34). A reasonable explanation is endothelial injury and neuroinflammation (35). Second, GFR estimations based on serum creatinine may be affected by muscle mass, creatine dietary supplements, or meat-based diet, and older people are more likely to have less than average muscle mass due to chronic disease, leading to low creatinine levels and underlying overestimation of GFR. Finally, albuminuria is an important risk factor for the development of both AD and mild cognitive impairment (MCI) (36). Moreover, albuminuria was also associated with temporal lobe atrophy and cortical thinning, independent of eGFR (37, 38). The mechanisms by which albuminuria is associated with brain atrophy may simply represent systemic endothelial dysfunction (39). Because most participants in our study were not from renal clinics, most of them lacked urinary albumin/creatinine ratio (UACR) data, and the prevalence of CKD may have been greatly underestimated.

We did not find eGFR <75 mL/min/1.73 m^2^ to be an independent risk factor for cognitive impairment although eGFR <75 mL/min/1.73 m^2^ was a risk factor for hippocampal atrophy. The possible reasons are as follows: First, the eGFR of the participants in our study is higher than other studies; second, hippocampal atrophy may precede cognitive impairment in the progression of the disease. Maybe it needs time for hippocampal atrophy to develop into cognitive impairment, and perhaps further follow-up will see the relationship between them.

Our study found LHCV <2,654 mm^3^ not RHCV was an independent risk factor for cognitive impairment and had the largest ROC value in ROC curve compared with age (Figure 4). HCV is a strong predictor of memory decline in MCI (40). A study found the involvement of white matter hyperintensities (WMH) and medial temporal lobe atrophy (MTA) were indicatives of AD pathology in cognitive dysfunction (41). So our study included white matter lesions as a covariate in the analysis of cognitive impairment and still found that decreased hippocampal volume was an independent risk factor for cognitive impairment. Wolf et al. (42) also found left-sided and posterior hippocampal measures were more responsible for cognitive impairment discrimination than right-sided and anterior measures. A study suggested that an increased APOE ɛ4 dose is associated with decreased effective brain-waste clearance, such as iron and β-amyloid, worsening the pathology of AD (43). These results suggest that APOE ε4 allele may lead to left hippocampal atrophy in patients with early-onset mild cognitive impairment, and the atrophy in some hippocampal subregions is more obvious (44). Logistic regression revealed that left hippocampal subiculum volume was a significant predictor of MCI conversion (45). Our study was in consistence with them. However, the cut-off value of LHCV was lower than other studies in China (46), may related to larger proportion of the elderly in our study.

This study has several strengths. There are few studies investigating the association of renal function, HCV, and cognitive function, especially in patients with normal renal function. Our study was a scarce clinical research in the nonrenal clinic population and had relatively large samples of nonrenal outpatients. Our results show that when eGFR <75 mL/min/1.73 m^2^, hippocampal atrophy may occur. Additionally, measurement of HCV provides a threshold value for the diagnosis of cognitive impairment, which is conducive to further research in the future.

Our study also had some limitations. First, due to the cross-sectional nature of this study, a future study with a larger cohort size is needed to determine causality or whether there is a causal relationship. Second, our hospital is a tertiary hospital with neurology as its dominant discipline. The participants were mostly from the outpatient department of neurology, which may have led to selection bias. Finally, the UACR test is lacking, which is another marker of kidney function in most outpatients in our study.

## Conclusion

We demonstrated that lower eGFR, even within normal renal function, are associated with HCV and cognition. LHCV <2,654 mm^3^ can predict cognitive impairment in outpatients.

## Data availability statement

The original contributions presented in the study are included in the article/supplementary material, further inquiries can be directed to the corresponding author.

## Ethics statement

The Research Ethics Boards at Xuanwu Hospital of Capital Medical University approved the study protocol (approval number: CTR-IPR-2019130). The studies were conducted in accordance with the local legislation and institutional requirements. Written informed consent for participation in this study was provided by the participants’ legal guardians/next of kin.

## Author contributions

L-YW: Data curation, Funding acquisition, Investigation, Software, Writing – original draft. Y-YL: Conceptualization, Formal analysis, Investigation, Methodology, Project administration, Supervision, Writing – review & editing. S-SZ: Data curation, Methodology, Project administration, Resources, Supervision, Validation, Writing – review & editing. Y-DC: Data curation, Formal analysis, Methodology, Project administration, Supervision, Writing – review & editing. JL: Supervision, Validation, Visualization, Writing – review & editing. A-HZ: Methodology, Supervision, Validation, Visualization, Writing – review & editing.
